# Identification of Prognostic Lactylation‐Related lncRNA Signatures and Immune Infiltration Characteristics in Gastric Cancer

**DOI:** 10.1155/ijog/7444301

**Published:** 2026-11-14

**Authors:** Jiaming Ge, Jie Xuan, Gaofeng Hu, Mengjia Li, Meigui Zhang, Yuanqiang Li, Jimin Su, Yaxin Zheng, Jinguo Hu, Xuefei Yu, Huangjie Zhang

**Affiliations:** ^1^ Department of Scientific Research Tongde Hospital of Zhejiang Province Hangzhou Zhejiang China zjtongde.com; ^2^ Department of Traditional Chinese Medicine Yincheng Sub-District Community Health Service Center Dexing Jiangxi China; ^3^ The First School of Clinical Medicine Zhejiang Chinese Medical University Hangzhou Zhejiang China zcmu.edu.cn; ^4^ Department of Pharmacy Hangzhou TCM Hospital Affiliated to Zhejiang Chinese Medical University Hangzhou Zhejiang China zcmu.edu.cn; ^5^ Department of Pharmacy Nanhu District People′s Hospital Jiaxing Zhejiang China

**Keywords:** gastric cancer, lactylation, long noncoding RNA, prognostic factor, prognostic model, tumor microenvironment

## Abstract

**Background:**

Lactylation, a novel epigenetic modification, plays a pivotal role in tumor metabolic reprogramming and regulation of the immune microenvironment. Long noncoding RNAs (lncRNAs) may be associated with gastric cancer (GC) progression and immune modulation through lactylation‐related processes.

**Methods:**

GC transcriptomic and clinical data were obtained from The Cancer Genome Atlas (TCGA). Lactylation‐related genes were retrieved from GeneCards. Lactylation‐related long noncoding RNAs (LLRs) were identified using Pearson correlation analysis. Prognosis‐related differentially expressed LLRs were screened using univariate Cox regression and least absolute shrinkage and selection operator (LASSO) regression analyses. Multivariable Cox regression analysis was performed to identify independent prognostic LLRs and construct a GC risk model. Patients were stratified into high‐ and low‐risk groups, followed by comparative analyses of Kaplan–Meier (K‐M) survival, the tumor microenvironment (TME), immune cell infiltration, tumor mutational burden (TMB), and immune evasion.

**Results:**

An 8‐LLR–based prognostic model was established to stratify patients with GC, and patients in the high‐risk group exhibited poorer overall survival. Receiver operating characteristic (ROC) curve analysis demonstrated moderate predictive performance, with a 5‐year area under the curve (AUC) of 0.727. Multivariable Cox regression analysis confirmed that the risk score was an independent prognostic factor. Significant differences in the TME, immune cell infiltration, TMB, and immune evasion were observed between the high‐ and low‐risk groups. Quantitative real‐time PCR (qRT‐PCR) confirmed differences in LLR expression between normal gastric epithelial cells and GC cell lines.

**Conclusion:**

This bioinformatic study explores the potential associations between LLRs, GC prognosis, and the tumor immune microenvironment. The 8‐LLR risk model demonstrated moderate predictive performance and provides an exploratory framework for GC risk stratification and the identification of candidate lncRNAs for further investigation.

## 1. Introduction

Gastric cancer (GC) is a prevalent and highly lethal malignancy worldwide, ranking among the leading causes of cancer‐related death [1–4]. Despite advances in surgical techniques, chemotherapy, and targeted therapies, most patients with GC are diagnosed at advanced stages and consequently have poor prognoses [5–7]. Therefore, the identification of sensitive and specific biomarkers and the establishment of reliable prognostic models are critically important for optimizing individualized treatment strategies and improving survival prediction.

Metabolic reprogramming has recently received increasing attention as a fundamental hallmark of cancer progression. Lactate, the end product of glycolysis, not only contributes to the remodeling of the tumor microenvironment (TME) but also regulates gene expression and signaling pathways through a novel posttranslational modification known as lactylation [8, 9]. This modification alters chromatin structure through histone modification, thereby influencing cancer cell proliferation, invasion, and immune evasion [10–13]. Long noncoding RNAs (lncRNAs) serve as essential epigenetic regulators and have been increasingly implicated in tumorigenesis, disease progression, and the orchestration of immune responses [14–17].

Recent bioinformatic studies have constructed lactylation‐associated gene‐ or lncRNA‐based prognostic signatures for multiple human malignancies [18, 19]. Nevertheless, few studies have specifically investigated lactylation‐related long noncoding RNAs (LLRs) in GC. Therefore, exploring the prognostic potential of LLRs in GC through transcriptome‐based computational analyses is of considerable value.

In this study, we integrated publicly available GC transcriptomic and clinical datasets to systematically identify LLRs. We subsequently developed a lncRNA‐based prognostic signature and rigorously evaluated its predictive performance for patient survival. Our bioinformatic analyses reveal potential correlative relationships involving LLRs in GC and provide candidate biomarkers for precision oncology research.

## 2. Materials and Methods

### 2.1. Data Acquisition

In this analysis, transcriptomic, clinical, and somatic mutation data from 412 gastric tumor samples and 36 normal gastric tissue samples were retrieved from The Cancer Genome Atlas (TCGA). Before prognostic model construction, five samples were excluded because of incomplete overall survival data, including missing survival time or vital status. Consequently, 407 patients with complete transcriptomic and survival data were included in subsequent survival‐related analyses. Perl scripts were used to distinguish mRNAs from lncRNAs. GeneCards was searched using the keyword “lactylation.” Genes with a GeneCards relevance score ≥ 0.2 were retained, and duplicate entries were removed. A total of 140 lactylation‐related genes were ultimately included for downstream analyses, and the complete gene list is provided in Table S1 [20].

### 2.2. Identification of LLRs

Pearson correlation analysis was conducted between lncRNAs and the 140 lactylation‐related genes in GC samples. Candidate LLRs were defined as lncRNAs exhibiting an absolute correlation coefficient (|*r*|) > 0.4 and *p* < 0.001 with at least one lactylation‐related gene. The differential expression of candidate LLRs between GC and normal tissues was subsequently evaluated using the Wilcoxon rank‐sum test. *p* values were adjusted for multiple comparisons using the Benjamini–Hochberg method, and LLRs with |log2FC| > 1.5 and a false discovery rate (FDR) < 0.05 were considered differentially expressed.

### 2.3. Construction of Prognostic Model and Risk Score Calculation

The expression profiles of the filtered LLRs were integrated with patient survival data, including survival time and vital status, to construct the dataset used for prognostic analysis. Univariate Cox proportional hazards regression analysis [20] was performed to identify LLRs significantly associated with overall survival (OS). To reduce dimensionality and prevent overfitting, least absolute shrinkage and selection operator (LASSO) penalized regression [21] was applied, followed by multivariable Cox regression analysis to identify lncRNAs retained in the final prognostic model.

The risk score for each patient was calculated as follows [22]:

Risk Score=∑Coefi×Expri,

where Coef_*i*_ is the regression coefficient, and Expr_*i*_ is the expression level of the ith lncRNA. Patients were subsequently categorized into high‐ and low‐risk groups according to the median risk score.

### 2.4. Model Validation, Independent Prognostic Analysis, and Nomogram Construction

Patients in the TCGA‐STAD cohort were randomly divided into training and testing cohorts at a 1:1 ratio. Risk scores were calculated independently for each sample in both cohorts. For cohort‐specific risk stratification, the median risk score derived from the training cohort was used as the predefined cutoff. This fixed cutoff was subsequently applied without modification to the testing cohort to classify patients into high‐ and low‐risk groups. Progression‐free survival (PFS) data were obtained from the TCGA clinical follow‐up dataset. The same 8‐LLR risk score model and predefined cutoff were applied to stratify patients for the PFS analysis. Cases with missing PFS time or PFS event status were excluded from the PFS survival analysis. Kaplan–Meier (K‐M) survival curves [23] were generated to compare OS between the high‐ and low‐risk groups, and statistical differences were assessed using the log‐rank test. Univariate and multivariable Cox regression analyses were conducted to determine whether the risk score remained an independent prognostic indicator after adjustment for clinical covariates. A prognostic nomogram incorporating the risk score and key clinicopathological factors was constructed to estimate 1‐, 3‐, and 5‐year survival probabilities. Calibration plots were used to evaluate the agreement between predicted and observed outcomes, whereas receiver operating characteristic (ROC) curves were used to assess the discriminative ability of the risk model.

### 2.5. Immune Infiltration and Tumor Mutation Analyses

To characterize the tumor immune microenvironment, ESTIMATE [24] was applied to calculate stromal, immune, and ESTIMATE scores for each risk group. The CIBERSORT algorithm [25] was used to deconvolute the relative proportions of 22 immune cell types, and differences in immune cell infiltration were compared between groups. Tumor Immune Dysfunction and Exclusion (TIDE) scores [26] were calculated to predict potential immune evasion and responsiveness to immune checkpoint blockade. Patients were stratified according to the median TMB, and survival analyses were performed to compare the high‐ and low‐TMB groups.

### 2.6. Gene Set Enrichment Analysis (GSEA) Enrichment and Drug Sensitivity Analysis

GSEA was performed using the clusterProfiler package to identify pathways enriched in each risk group. The R package pRRophetic was used to predict the half‐maximal inhibitory concentration (IC50) of candidate compounds. The gene expression matrix of TCGA GC samples was used as input. Prediction models were trained using the GDSC drug sensitivity dataset with default parameters to estimate the IC50 values of individual agents for each patient.

### 2.7. Quantitative Real‐Time PCR (qRT‐PCR) Analysis

Total RNA was extracted from cells, followed by reverse transcription using the Strand cDNA Synthesis Kit (Invitrogen, Carlsbad, United States). Each qRT‐PCR experiment was performed using three independent biological replicates, with three technical replicates for each sample. The mean Ct values of the technical replicates were used for subsequent analysis. 18S rRNA served as the internal normalization control. Relative RNA expression was calculated using the 2^−*ΔΔ*Ct^ method, and the Wilcoxon rank‐sum test was used for statistical comparisons between cell groups.

Primer sequences used in this study are provided in Table 1.

**Table 1:** Primer sequences for qRT‐PCR.

Gene symbol	Forward primer (5 ^′^ → 3 ^′^)	Reverse primer (5 ^′^‐3 ^′^)
AC079584.2	AGCATGATTGGCATAAATGTGGC	AGCAGATAGCATCCATTCAGGG
LINC01940	AGCTGTGAGACAACCGAAGG	CAAAGCCTTCCCTTTGAGCG
AC010275.1	CCACTCCTGATTGACCACCC	CGCGGTAGTTTTTCGATTACCA
CYMP‐AS1	ACGCCGTCAATGATGACACT	CCCTCCCTACTGCAGATCCT
GAS1RR	AAGGGGGCAACTGTATACGC	GGAGGTGCTTGATCACTGGT
HAGLR	CACCAACCACGGACATTCCT	GGGAAGAGCCAAGTCAGACG
VPS9D1‐AS1	CGCACATGGTCCTGTTCGTA	CCTTTCTCCCCAGGAGGTTT
LINC00449	GGAAGACCCGAGCATACCTG	CGGACACCGTCTCACATCAT
18S	CCTTTGCCATCACTGCCATT	CACACGTTCCACCTCATCCTC

### 2.8. Statistical Analysis

All analyses were performed using R Version 4.4.1 and GraphPad Prism Version 10.3.1. Cox regression models, nomograms, and K‐M survival curves were generated using the survival and survminer packages. Differential expression comparisons were performed using the Wilcoxon rank‐sum test, LASSO regression was conducted using glmnet, and prognostic accuracy was assessed using survival ROC. A two‐tailed *p* value below 0.05 was deemed significant.

## 3. Results

### 3.1. Identification of LLRs and Construction of a Prognostic Model

GC transcriptomic data were obtained from TCGA, and customized Perl scripts were used to distinguish mRNAs from lncRNAs. A total of 140 lactylation‐related genes were curated from GeneCards to construct a lactylation‐specific expression matrix. Pearson correlation analysis (|*r*| > 0.4, *p* < 0.001) identified associations between these genes and 1428 lncRNAs, generating a coexpression network comprising 52 lactylation‐related genes (Figure 1A). Differential expression analysis identified 410 upregulated and 30 downregulated lncRNAs in tumor tissues compared with normal tissues (Figure 1B), and the Top 50 upregulated and downregulated lncRNAs are shown in a heatmap (Figure 1C). Prognostic screening of the 440 candidate lncRNAs identified 18 lncRNAs significantly associated with overall survival, including two protective factors and 16 risk factors. Sequential LASSO regression and multivariable Cox regression analyses further refined the panel to eight key LLRs (Figure 1E,F): AC079584.2, LINC01940, VPS9D1‐AS1, AC010275.1, CYMP‐AS1, GAS1RR, HAGLR, and LINC00449. These LLRs served as the basis for the prognostic risk signature. The regression coefficients, hazard ratios (HRs), 95% confidence intervals (CIs), and *p* values of the 8‐LLRs included in the final multivariable Cox model are summarized in Table 2.

**Figure 1:**
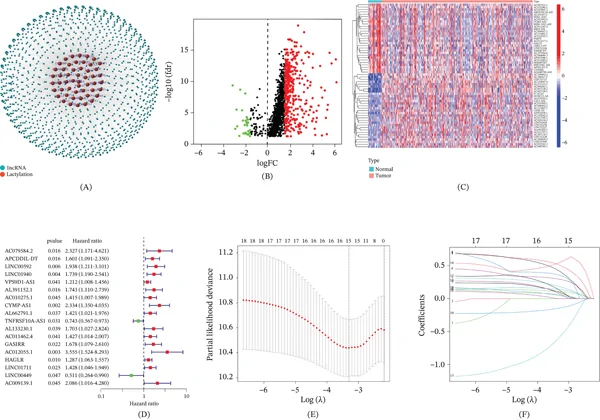
Construction of a prognostic model based on LLRs in GC. (A) Coexpression network of LLRs. (B) Upregulated (red) and downregulated (green) LLRs in tumor tissues compared with normal tissues. (C) Heatmap of differentially expressed LLRs in tumor and normal samples. (D) Univariate Cox regression analysis of LLRs associated with OS. (E,F) LASSO regression analysis identifying the optimal *λ* value and corresponding coefficients for the prognostic model.

**Table 2:** Multivariable Cox regression parameters of the eight lactylation‐related lncRNAs included in the prognostic signature.

LLR	Coefficient	HR	95% CI	*p*
AC079584.2	0.850	2.339	1.143–4.788	0.020
LINC01940	0.569	1.767	1.204–2.593	0.004
VPS9D1‐AS1	0.364	1.439	1.168–1.773	< 0.001
AC010275.1	0.458	1.581	1.061–2.356	0.024
CYMP‐AS1	0.858	2.358	1.318–4.218	0.004
GAS1RR	0.707	2.028	1.199–3.430	0.008
HAGLR	0.238	1.269	1.025–1.570	0.029
LINC00449	−1.123	0.325	0.152–0.695	0.004

### 3.2. Internal Validation of the LLR‐Based Prognostic Model

A total of 407 patients with GC were randomly divided into training and testing cohorts. The median risk score calculated from the training cohort was used as the fixed cutoff to stratify both the training and testing cohorts into high‐ and low‐risk groups. K‐M survival analysis demonstrated significantly better survival among low‐risk patients in the total cohort (Figure 2A), training cohort (Figure 2D), and testing cohort (Figure 2G). ROC curve analysis was subsequently performed to evaluate the predictive accuracy of the model, yielding AUC values of 0.804, 0.632, and 0.704 in the training, testing, and total cohorts, respectively (Figure 2B,E,H). Heatmaps illustrated the differential expression patterns of the eight lncRNAs in the training, testing, and total cohorts (Figure 2C,F,I). These findings indicate that the model retained prognostic discriminatory ability in both subsets of the TCGA‐STAD cohort, although the magnitude of discrimination varied among subsets.

**Figure 2:**
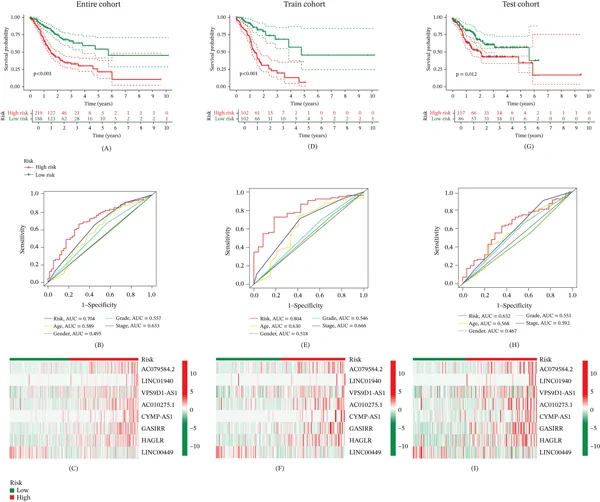
Validation of the prognostic model. (A,D,G) K‐M survival analysis of GC patients. (B,E,H) ROC curves showing the AUC values of the risk score and clinical characteristics in the entire cohort, training set, and testing set. (C,F,I) Heatmaps showing the expression patterns of the 8‐LLRs in two risk groups from the entire cohort, training set, and testing set.

### 3.3. Independent Prognostic Value of the LLR‐Based Risk Model

Univariate Cox regression analysis showed that age (HR = 1.024, *p* = 0.004), tumor grade (HR = 1.404, *p* = 0.042), tumor stage (HR = 1.591, *p* < 0.001), and risk score (HR = 1.086, *p* < 0.001) were significantly associated with prognosis (Figure 3A). Multivariable Cox regression analysis further confirmed that age (HR = 1.036, *p* < 0.01), tumor stage (HR = 1.755, *p* < 0.001), and risk score (HR = 1.098, *p* < 0.001) were independent prognostic predictors (Figure 3B). Time‐dependent ROC analysis yielded AUC values of 0.678, 0.704, and 0.727 for predicting 1‐, 3‐, and 5‐year OS, respectively (Figure 3C), indicating moderate time‐dependent discriminatory performance. The K‐M survival curve demonstrated a significant difference in PFS between the high‐ and low‐risk groups (*p* = 0.012; Figure 3D), indicating that the risk score provides prognostic stratification for patients.

**Figure 3:**
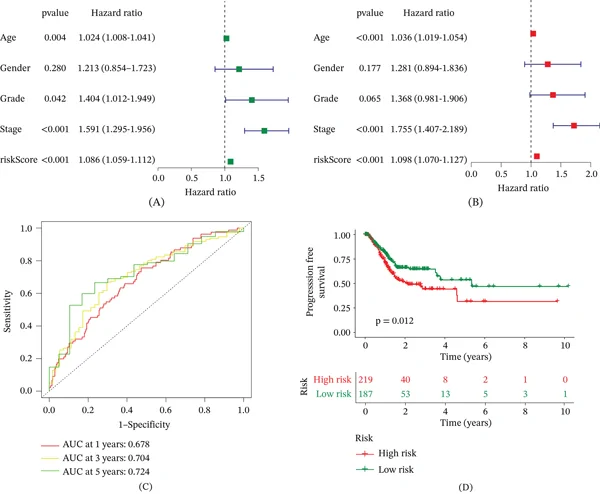
Evaluation of the independent prognostic value of the risk model. (A,B) (A) Univariate and (B) multivariable Cox regression analyses demonstrating the independent prognostic value of the risk score. (C) Time‐dependent ROC curves showing AUC values for 1‐, 3‐, and 5‐year OS. (D) K‐M curve of progression‐free survival stratified by risk score. The bold value is the log‐rank p value (*p* =0.012), indicating a significant difference in progression‐free survival between the high‐risk and low‐risk groups. The bold formatting is intended to highlight the statistical result.

### 3.4. Construction and Validation of a Nomogram

A nomogram integrating age, sex, TNM stage, tumor grade, and the LLR‐based risk score was constructed to predict 1‐, 3‐, and 5‐year survival probabilities (Figure 4A). The concordance index (C‐index) demonstrated comparable or slightly improved performance of the combined model compared with individual clinical factors (Figure 4B), whereas calibration curves showed moderate to good agreement between predicted and observed outcomes (Figure 4C). These findings suggest that the nomogram incorporating the LLR‐based risk score and clinical characteristics provides a quantitative reference for estimating OS in patients with GC.

**Figure 4:**
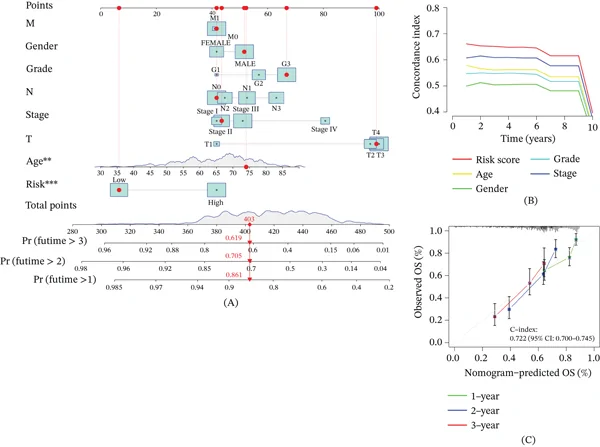
Construction and validation of the nomogram model. (A) A nomogram predicting 1‐, 3‐, and 5‐year OS in GC patients. (B) C‐index comparing the predictive performance of risk score with clinical features. (C) Calibration curves for 1‐, 3‐, and 5‐year OS, showing good agreement between predicted and observed outcomes. ∗∗*p* < 0.01, ∗∗∗*p* < 0.001. The red values in Figure 4A illustrate an example of how to interpret the nomogram. For a representative patient with a total score of 403 points, the predicted overall survival probabilities at 1, 2, and 3 years are 0.861 (86.1%), 0.705 (70.5%), and 0.619 (61.9%), respectively. These values are provided for illustrative purposes to demonstrate how the nomogram can be used to estimate individual survival probabilities.

### 3.5. TME and Immune Cell Infiltration Analysis

To explore the association between the risk model and the TME, the ESTIMATE algorithm was applied. The high‐risk group exhibited significantly higher immune, stromal, and ESTIMATE scores than the low‐risk group (Figure 5A–C). CIBERSORT deconvolution analysis demonstrated increased naïve B‐cell infiltration in high‐risk samples, whereas neutrophils and plasma cells were more abundant in low‐risk samples (Figure 5D). The immune cell composition across the two risk groups is presented in the heatmap (Figure 5E). These findings indicate that the LLR‐based risk groups are associated with distinct TME and immune cell infiltration profiles.

**Figure 5:**
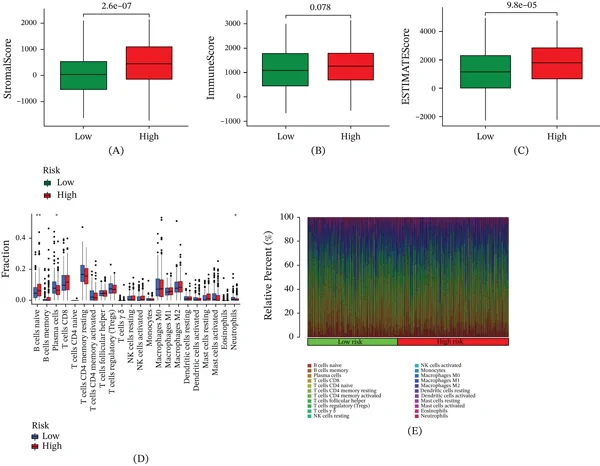
Tumor microenvironment and immune cell infiltration analysis. (A–C) Comparisons of stromal, immune, and ESTIMATE scores between the two risk groups. (D) Immune cell infiltration landscape in GC samples. (E) Heatmap of relative proportions of immune cell types. ∗*p* < 0.05, ∗∗*p* < 0.01, ∗∗∗*p* < 0.001.

### 3.6. Somatic Mutation and Immunotherapy Response Analysis

Somatic mutation data were visualized using waterfall plots. TTN, MUC16, LRP1B, and ARID1A mutations were more prevalent in low‐risk tumors, whereas TP53 alterations predominated in the high‐risk group (Figure 6A,B). Low‐risk cases also exhibited higher TMB (Figure 6C), and patients in the high‐TMB subgroup showed superior survival outcomes (Figure 6D). Combined TMB and risk score analysis identified the high‐TMB/low‐risk group as having the most favorable prognosis (Figure 6E). In addition, low‐risk patients had lower TIDE scores (Figure 6F). These computational predictions suggest that low‐risk patients may have a more favorable tumor immune microenvironment and may potentially respond better to immune checkpoint inhibitors. However, these are in silico predictions only. Immunotherapy response predictions should be regarded as hypothesis‐generating and require confirmation in clinical cohorts treated with immune checkpoint inhibitors.

**Figure 6:**
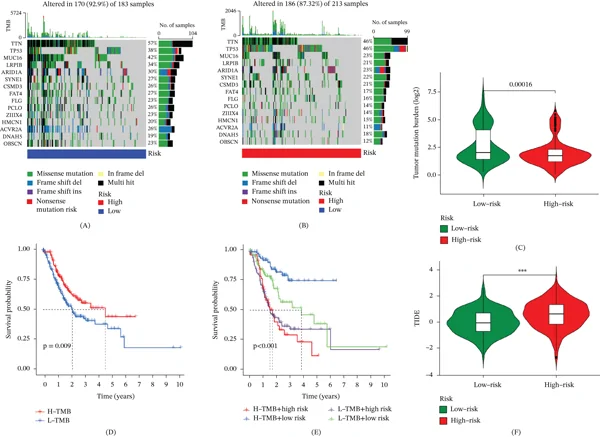
Somatic mutation profiles and immune evasion analysis between two risk groups. (A,B) Waterfall plots of somatic mutation profiles in (A) low‐risk and (B) high‐risk groups. (C) Comparison of TMB scores. (D) K‐M survival analysis of high‐TMB versus low‐TMB groups. (E) Combined survival analysis based on TMB status and risk groups. (F) TIDE score comparison between the two risk groups. ∗∗∗p < 0.001.

### 3.7. GSEA and Drug Sensitivity Analysis of the Lactylation‐Related Prognostic Model

GSEA revealed that the high‐risk group was primarily enriched in pathways related to cell adhesion molecules (CAMs), dilated cardiomyopathy, focal adhesion, and hypertrophic cardiomyopathy (HCM) (Figure 7A). In contrast, the low‐risk group was predominantly enriched in amino sugar and nucleotide sugar metabolism, glycerolipid metabolism, oxidative phosphorylation, and ribosome‐related pathways (Figure 7B). These results provide a hypothesis that tumors in high‐risk patients may be enriched in pathways biologically associated with tumor invasion and metastasis. Similarly, the enrichment of metabolic pathways in the low‐risk group suggests, as a hypothesis, that low‐risk tumors may retain more normal metabolic features. Drug sensitivity analysis suggested that high‐risk patients tended to have lower predicted IC50 values for dasatinib and alpelisib, whereas low‐risk patients exhibited lower predicted IC50 values for savolitinib and erlotinib (Figure 7C–F). These findings computationally predict potential differences in drug responses between the two risk groups. However, the IC50 values estimated using pRRophetic are based on in silico predictions. Therefore, their potential utility in guiding personalized treatment remains exploratory and requires further validation in clinical pharmacogenomic studies.

**Figure 7:**
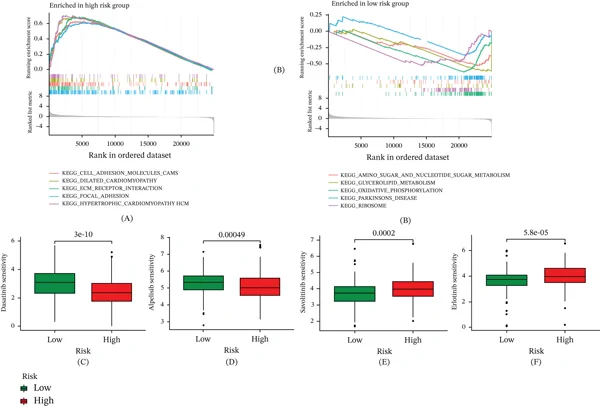
The results of GSEA enrichment and drug sensitivity analysis. (A,B) Significantly enriched KEGG pathways in two risk groups. (C–F) Predicted sensitivity to dasatinib, alpelisib, savolitinib, and erlotinib in the two risk groups.

### 3.8. Validation of LLRs Expression in GC Cell Lines

To validate the expression of the 8‐LLRs, qRT‐PCR was performed in normal gastric epithelial cells (GES‐1) and GC cell lines (AZ‐521 and MKN‐45). As shown in Figure 8, the expression levels of AC079584.2, LINC01940, VPS9D1‐AS1, AC010275.1, CYMP‐AS1, GAS1RR, HAGLR, and LINC00449 differed significantly between GC cell lines and normal gastric epithelial cells. These findings indicate that these lncRNAs are differentially expressed between cancer and normal cells and warrant further investigation.

**Figure 8:**
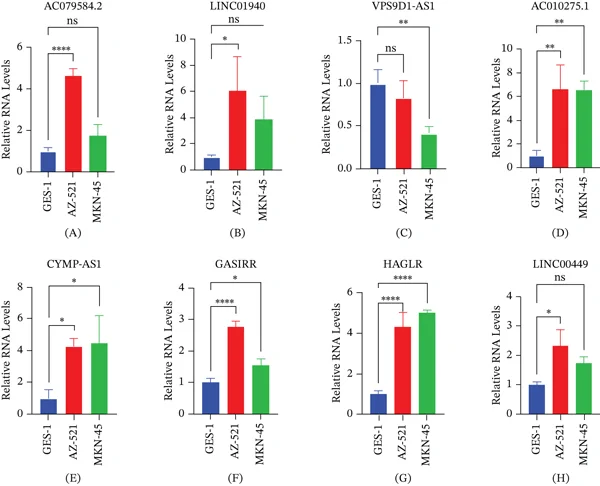
Expression validation of core LLRs. (A–H) qRT‐PCR was performed to validate the differential expression of core LLRs in normal gastric epithelial cell (GES‐1) and GC cell lines (AZ‐521 and MKN‐45).  ^∗^ *p* < 0.05,  ^∗∗^ *p* < 0.01,  ^∗∗∗^ *p* < 0.001, and  ^∗∗∗∗^ *p* < 0.0001; ns: not significant.

## 4. Discussion

Tumor initiation and progression involve complex genetic alterations and signaling network dysregulation [27–29]. In recent years, accumulating evidence has highlighted the dual role of lactate as both a metabolic substrate and a signaling metabolite in cancer biology [30, 31]. In addition to serving as an energy source for glycolytic tumor cells, lactate‐mediated protein lactylation can remodel chromatin architecture and transcriptional programs [32, 33], thereby promoting oncogenic transformation, proliferation, and metastatic dissemination. Concurrently, elevated lactate levels within the TME impair the functions of cytotoxic T cells and natural killer cells, facilitating immune evasion and tumor progression [34, 35]. Zhang et al. [33] demonstrated that lactate can contribute to the regulation of gene expression through histone lactylation. Lactylation can promote macrophage polarization toward the M2 phenotype, thereby suppressing immune responses within the TME [36]. However, the associations between LLRs and GC have not yet been investigated. Therefore, the present study explored the potential role of lactylation in GC by identifying LLRs and constructing a prognostic model based on eight key lncRNAs: AC079584.2, LINC01940, VPS9D1‐AS1, AC010275.1, CYMP‐AS1, GAS1RR, HAGLR, and LINC00449.

Among these LLRs, VPS9D1‐AS1 has been associated with the progression of colorectal cancer, lung adenocarcinoma, and endometrial cancer [37–39], and its expression in GC tissues is significantly associated with tumor size and TNM stage [40]. CYMP‐AS1 and LINC00449 have also been identified as promising prognostic biomarkers for GC and have been incorporated into predictive models [41–43], whereas HAGLR regulates GC progression through modulation of the miR‐20a‐5p/E2F1 axis [44–46]. Notably, four of the eight prognostic LLRs identified in the present study have not previously been linked to GC. We evaluated the prognostic performance of the 8‐lncRNA signature in the training and testing cohorts. High‐risk patients exhibited poorer survival outcomes than low‐risk patients, suggesting that the model could stratify patients according to prognosis. Moreover, the risk score remained independently associated with overall survival after adjustment for the included clinical variables. We further developed a composite nomogram integrating the risk score with key clinicopathological variables to estimate 1‐, 3‐, and 5‐year survival probabilities. Notably, AUC values varied across the training, testing, and entire cohorts, indicating differences in the magnitude of discriminatory performance among data subsets. Nevertheless, the signature retained prognostic stratification ability within the TCGA cohort. Further evaluation in independent cohorts will be important to assess its generalizability.

The TME is a key determinant of GC progression and therapeutic response [47–50], with lactate playing a central role in TME remodeling [51, 52]. Previous studies have shown that lactate affects multiple TME components through extracellular matrix (ECM) remodeling, cancer‐associated fibroblast (CAF) activation, and immune suppression, thereby promoting tumor progression [51–53]. In the present study, stromal, immune, and ESTIMATE scores were all higher in the high‐risk group, with significant differences observed in stromal and ESTIMATE scores. These findings suggest that nontumor components may be more enriched in the TME of high‐risk patients, potentially enhancing stromal barriers and promoting an immunosuppressive microenvironment, thereby affecting patient prognosis. Our computational analyses identified associations between the LLR‐derived risk score, TME characteristics, and immune cell infiltration profiles. These observational findings are strictly correlative and do not establish that the identified lncRNAs influence or reshape the TME. Further experimental and clinical studies are required to clarify the biological basis of these associations.

Regarding immune cell infiltration, the proportions of plasma cells and neutrophils were significantly higher in the low‐risk group. Plasma cells mediate humoral immunity and have been associated with favorable prognoses in multiple solid tumors [54, 55]. Neutrophils exhibit context‐dependent functions within the TME. Although lactate can induce their protumor polarization, the increased neutrophil abundance observed in the low‐risk group may reflect preserved antitumor innate immunity [56, 57]. Notably, the results generated by ESTIMATE and CIBERSORT are observational and cannot demonstrate causality. Whether the eight identified lncRNAs actively regulate the immune microenvironment remains to be experimentally validated.

Because these lncRNAs were screened according to their correlations with lactylation‐related genes, potential mechanistic hypotheses can be proposed. Coexpression analysis revealed close correlations between the signature LLRs and lactylation‐regulatory genes, suggesting that these lncRNAs may indirectly modulate lactate‐driven epigenetic reprogramming in GC [10, 33]. Consistent with the GSEA results, the high‐risk signature was associated with enrichment of cell adhesion and focal adhesion pathways, which are linked to tumor invasion and metastasis. In contrast, metabolic pathways, including oxidative phosphorylation and glycerolipid metabolism, were enriched in the low‐risk group. Collectively, these bioinformatic observations provide hypothesis‐generating evidence of potential links among LLRs, lactylation‐associated signaling, TME features, and GC progression. However, direct experimental evidence is required to elucidate how these lncRNAs interact with lactylation‐related molecules and exert their biological effects.

Currently, TMB has important value in predicting immunotherapy responses, guiding treatment decisions, assessing prognosis, and facilitating cancer research, making it a key biomarker in precision medicine [58, 59]. In the present study, TMB was significantly higher in the low‐risk group, and patients with high TMB exhibited better overall survival than those with low TMB. Furthermore, patients in the low‐risk/high‐TMB group had the most favorable prognosis. In addition, TIDE scores were significantly lower in the low‐risk group. Previous studies have demonstrated that high TMB reflects a greater number of somatic mutations within tumors, which may generate more neoantigens and enhance immune recognition and responses. This may result in stronger antitumor immunity and improved prognosis. Moreover, patients with high TMB have been shown to respond more favorably to immune checkpoint inhibitors, suggesting that they may derive greater benefit from immunotherapy [60–62]. These observations are consistent with our TIDE results, indicating that low‐risk patients may exhibit a better response to immunotherapy. However, this prediction requires further validation in real‐world GC cohorts receiving immune checkpoint inhibitors.

Despite these findings, several limitations should be acknowledged. First, the absence of external validation remains the most important limitation of this study. Prognostic validation was confined to the TCGA‐STAD cohort because we were unable to identify a suitable external dataset containing expression data for all eight LLRs and the required survival information. Consequently, the generalizability of the 8‐LLR signature across populations from different geographic regions, ethnic backgrounds, and treatment eras remains uncertain. Future validation in independent, multicenter cohorts is therefore essential to confirm the prognostic performance and clinical utility of this signature. Second, the mechanisms through which these lncRNAs influence gastric carcinogenesis remain unexplored. Apart from expression validation in cell lines, no functional assays were conducted. Future studies should incorporate comprehensive in vitro and in vivo experiments to validate the biological roles and therapeutic potential of these lactylation‐associated lncRNAs. Third, the notable decline in AUC from the training cohort (0.804) to the testing cohort (0.632) suggests that the model may be overfitted to the training data. This is a recognized limitation of prognostic signatures developed using a single, relatively small cohort. Bootstrap correction, repeated cross‐validation, and external validation in independent cohorts will be necessary to more rigorously assess the generalizability and robustness of this signature. In addition, several important clinical and molecular covariates, including detailed T/N/M subclassification, chemotherapy and radiotherapy history, Epstein–Barr virus (EBV) infection status, and microsatellite instability (MSI) status, had substantial missing values in the TCGA‐STAD dataset and therefore could not be included in multivariable Cox regression adjustment. This limitation may have resulted in residual confounding.

In conclusion, we developed an LLR‐based prognostic model that showed potential for stratifying patients with GC according to survival risk. The risk score was also associated with TME characteristics, immune cell infiltration, and somatic mutation profiles. These findings provide a hypothesis‐generating framework for further investigation of LLRs in GC and warrant further evaluation for their potential clinical utility. However, because the model has not been validated in an independent cohort, its generalizability and clinical utility require confirmation in external multicenter cohorts and prospective studies.

## Author Contributions

Jiaming Ge: writing—original draft, data curation, and validation. Jie Xuan: investigation, and data curation. Gaofeng Hu: methodology and data curation. Mengjia Li, Yuanqiang Li, and Meigui Zhang: data curation and validation. Jimin Su and Yaxin Zheng: visualization. Jinguo Hu: writing—original draft and project administration. Xuefei Yu: writing—review and editing and supervision. Huangjie Zhang: conceptualization, writing—review and editing, and supervision. Jiaming Ge, Jie Xuan, and Gaofeng Hu have contributed to the work equally and should be regarded as co‐first authors.

## Funding

This study was supported by the Traditional Chinese Medicine Science and Technology Project of Zhejiang Province (2023ZR065).

## Ethics Statement

All data used in this study were obtained from the publicly available, deidentified TCGA dataset. Therefore, no additional ethics approval or informed consent was required for this secondary analysis.

## Conflicts of Interest

The authors declare no conflicts of interest.

## Supporting information


**Supporting Information** Additional supporting information can be found online in the Supporting Information section. Table S1 presents the GeneCards‐derived list of candidate genes included in this study. The table provides the gene symbol, gene description, gene category, UniProt ID, GeneCards identifiers, and scores (including Gifts and relevance score), as well as direct links to the corresponding GeneCards entries.

## Data Availability

The patient‐level transcriptomic, clinical, and somatic mutation data analyzed in this study are publicly available from The Cancer Genome Atlas Stomach Adenocarcinoma (TCGA‐STAD) project through the NCI Genomic Data Commons Data Portal (project ID: TCGA‐STAD; https://portal.gdc.cancer.gov/projects/TCGA-STAD). Lactylation‐related genes were retrieved from GeneCards (https://www.genecards.org/), and the complete gene list is provided in Table S1. The drug sensitivity reference data underlying the pRRophetic analysis were derived from the Genomics of Drug Sensitivity in Cancer (GDSC) database (https://www.cancerrxgene.org/). The data generated by the qRT‐PCR experiments are available from the corresponding authors upon reasonable request.
